# Online surveillance of media health event reporting in Nepal: digital disease detection from a One Health perspective

**DOI:** 10.1186/s12914-017-0134-2

**Published:** 2017-09-21

**Authors:** Jessica S. Schwind, Stephanie A. Norman, Dibesh Karmacharya, David J. Wolking, Sameer M. Dixit, Rajesh M. Rajbhandari, Sumiko R. Mekaru, John S. Brownstein

**Affiliations:** 1 Augusta University, Augusta, GA USA; 2 Marine-Med, Bothell, Washington, USA; 3 Center for Molecular Dynamics-Nepal, Kathmandu, Nepal; 4 One Health Institute, University of California, Davis, California, USA; 5 HealthMap, Boston Children’s Hospital, Boston, MA USA; 6 Epidemico, Inc., Boston, MA USA; 7 Jiann-Ping Hsu College of Public Health, Georgia Southern University, P.O. Box 8015, 30460 Statesboro, GA USA

**Keywords:** Media, Nepal, Digital disease detection, Internet health reports, One health

## Abstract

**Background:**

Traditional media and the internet are crucial sources of health information. Media can significantly shape public opinion, knowledge and understanding of emerging and endemic health threats. As digital communication rapidly progresses, local access and dissemination of health information contribute significantly to global disease detection and reporting.

**Methods:**

Health event reports in Nepal (October 2013–December 2014) were used to characterize Nepal’s media environment from a One Health perspective using HealthMap - a global online disease surveillance and mapping tool. Event variables (location, media source type, disease or risk factor of interest, and affected species) were extracted from HealthMap.

**Results:**

A total of 179 health reports were captured from various sources including newspapers, inter-government agency bulletins, individual reports, and trade websites, yielding 108 (60%) unique articles. Human health events were reported most often (*n* = 85; 79%), followed by animal health events (*n* = 23; 21%), with no reports focused solely on environmental health.

**Conclusions:**

By expanding event coverage across all of the health sectors, media in developing countries could play a crucial role in national risk communication efforts and could enhance early warning systems for disasters and disease outbreaks.

## Background

Traditional media sources such as television, radio, magazines, newspapers and other printed forms of communication, as well as the internet, are crucial in the distribution of health information across all regions of the globe. Media, particularly domestic media published in-country, can play an important role in shaping the public’s opinion, knowledge and understanding of emerging health threats, current endemic diseases, and natural disasters such as earthquakes [1]. In general, studies have shown that the public obtains or seeks out more information concerning health risk and hazard from the media than they receive from their doctors, family and friends [2, 3]. Therefore, the reporting of any health-related events through mass media is a significant part of risk communication plans in many countries [4, 5]. Thus, health event reporting to the public must be accurate and consistent with regards to level of comprehension and detail; failure to do so may increase alarm or contribute to actions detrimental to resolution of the health event in question [6, 7]. This information flow cannot happen effectively and efficiently if the domestic media environment does not report on health-related events or reports inaccurate or incomplete information. Because it is not always health professionals who convey this information directly to the population, domestic media becomes an important tool to aid in the proper dispersion of accurate information to all stakeholders [8, 9].

With technological advancements, especially in developing countries, domestic sources of information play an increasingly important role in global disease surveillance [10–12]. Health event reporting of local events have been shown to be a crucial part of global early warning systems for digital disease detection and response, often providing key information regarding outbreaks well before traditional methods of surveillance [13–16]. However, past studies have shown that the type of local information presented on the global stage may not be as robust or comprehensive as desired [11, 12, 17]. While the reasons for this may vary across countries, research indicates that this lack of health event reporting exists not only in developing countries, but also in more established media environments [11, 17, 18].

In addition to human health event reporting, animal and environmental health concerns are also important to communicate through the media as they provide a critical link between professionals who focus on research, policy and practice in these fields and the communities who are inextricably related to both as a part of the overall ecosystem. By working to bring diverse sectors and organizations together for collaborative engagement, the interdisciplinary One Health approach helps promote cooperation and collaboration among human, animal and ecosystem health professionals [19, 20]. Currently, the One Health approach is the guiding force in several leading research initiatives, scientific conferences/programs and surveillance systems aimed at better understanding the linkages across all areas of health [20–23]. However, examining health event reporting from a One Health perspective is notably sparse and can be problematic as health-related events pertaining to animal and environmental health may go unnoticed or underreported due to lack of media coverage or detection when compared to human health reporting [22].

This study focuses on the reporting of health events in Nepal in order to characterize Nepal’s media environment from a One Health perspective using a global online disease surveillance and mapping tool. Nepal is a land-locked country situated between India and China in South Asia. After the transition from an autocratic government to a democracy in 1990 and the introduction of the New Communication Policy in 1992, the number of electronic and print media has steadily increased throughout the country [24]. Currently, there is a thriving presence of various forms of media outlets in Nepal, including radio, television, newspaper and magazines. Additionally, online (internet) based news portals are also increasing as the percentage of the population with internet connectivity expands [24]. Despite this media growth, the number of domestic health events reaching global media outlets may still be limited. The problem may be exacerbated if global media only reports in a common internet language like English, ignoring reports in local languages/dialects.

Prompt and accurate health event reporting in Nepal could be crucial in the early recognition of disease outbreaks as Nepal is considered a global hotspot zone for disease emergence [25]. In urban areas, high population density of both humans and animals in market areas, coupled with poor sanitation, make this a prime interface of potential disease transmission [26]. In addition, many rural villagers raise poultry and other domesticated animals for income and consumption, typically keeping them close to or inside the home. Because of the increased intensity of animal production across the country, only mass animal die-offs, or mortalities involving species of relatively great economic importance, may tend to be the animal health events deemed newsworthy. In order to better understand the status of Nepali health event reporting and its contribution to the global digital disease surveillance systems across human, animal and environmental health sectors, the main objective of this research was to apply the One Health perspective in characterizing online media health event reporting in Nepal.

## Methods

### Data collection

For the purposes of this study, a health event was defined as a disease or death whose occurrence either prompted epidemiologic studies or served as a cautionary indication that the quality of preventive and/or therapeutic medical care may need to be enhanced. All health event reports were constructed using information captured through the HealthMap online surveillance system [16, 27]. HealthMap is an internet-based surveillance system that aggregates multiple web-based data sources, such as news aggregation reports, online newspaper feeds, governmental and non-governmental bulletins and other online surveillance system notifications (e.g. ProMED-mail), and marks the locations on a map for a visual display of nearby outbreaks or local alerts globally. HealthMap supports 15 languages (including all official UN languages). All health reports related to events occurring in Nepal in HealthMap from October 2013 through December 2014 were included in the study. The catchment period included 14 months to provide an opportunity to detect infectious diseases that may have a seasonal component*.*

### Variable construction

For each health event report received through HealthMap, the following variables were defined: 1) *Region*, defined as the Development Regions of Nepal whereby the country is divided into five administrative divisions, or regions – Farwestern, Midwestern, Western, Central, Eastern, Multiple (for events covering more than one region), or Unspecified (regional information could not be determined even after thorough investigation of the report); 2) *Zone*, which characterizes any of the 14 administrative subdivisions within the regions, in addition to Multiple and Unspecified; 3) the *Report topic*, which best described the applicable element of the One Health approach (Human, Animal, Environment) covered by the report; 4) the *Report nature* that corresponded to the type of information relayed by the report – either *active* (ongoing disease or health event), *warning* (risk of disease or health event occurring, usually in response to an environmental event such as flooding), or *informational* (no ongoing disease or risk of a disease or health event); 5) *Unique event* (yes or no), the event reported was a new, previously unreported health event in the database; 6) *Unique report* (yes or no) whether the article was a unique (not duplicate) report in HealthMap; 7) *Media type* described the type of media medium such as: newspaper; government agency press release or bulletin; individual (typically a medical professional or researcher); or trade website (such as food industry stakeholders); 8) *Nationality* of the report source, classified as domestic (Nepal) or international (health event was reported from a source primarily located outside of Nepal); and 9) *Primary species* involved in the health event, designated as humans, wildlife or livestock (cattle, oxen, yaks, or goats) and poultry (unspecified domestic birds, chickens and poultry were grouped together). In order to identify unique events, characteristics such as location, date, and reported signs and symptoms were cross-referenced with similar reports. If reports matched on these features, but were unique in words, it was considered to be two separate reports writing about the same event.

### Data analysis

Raw counts and percentages were used to tabulate the number of unique health reports in Nepal (duplicate reports were excluded), as well as aforementioned event characteristics. A contingency table analysis using Fisher’s exact tests were used to compute summary statistics for the association between event characteristics and source origination (international versus domestic). All analyses were conducted using Stata™ (version 10.0, StataCorp, College Station, TX), and a *p*-value of ≤0.05 was regarded as significant. ArcGIS (version 10.3.1, ESRI, Redlands, CA) was utilized to construct a map of Nepal that detailed the number of unique health reports by Development Region.

## Results

A total of 179 health reports from Nepal were extracted from HealthMap, consisting of 50 unique events, from October 2013 through December 2014. After removing the duplicate reports, 108 (60.3%) were identified as unique reports and thus retained for analysis. Duplicate reports were re-posts of the original report through social media outlets and global electronic health event reporting systems such as GoogleNews (*n* = 27), ProMED-mail (*n* = 24), and Twitter (*n* = 20).

**Fig. 1:**
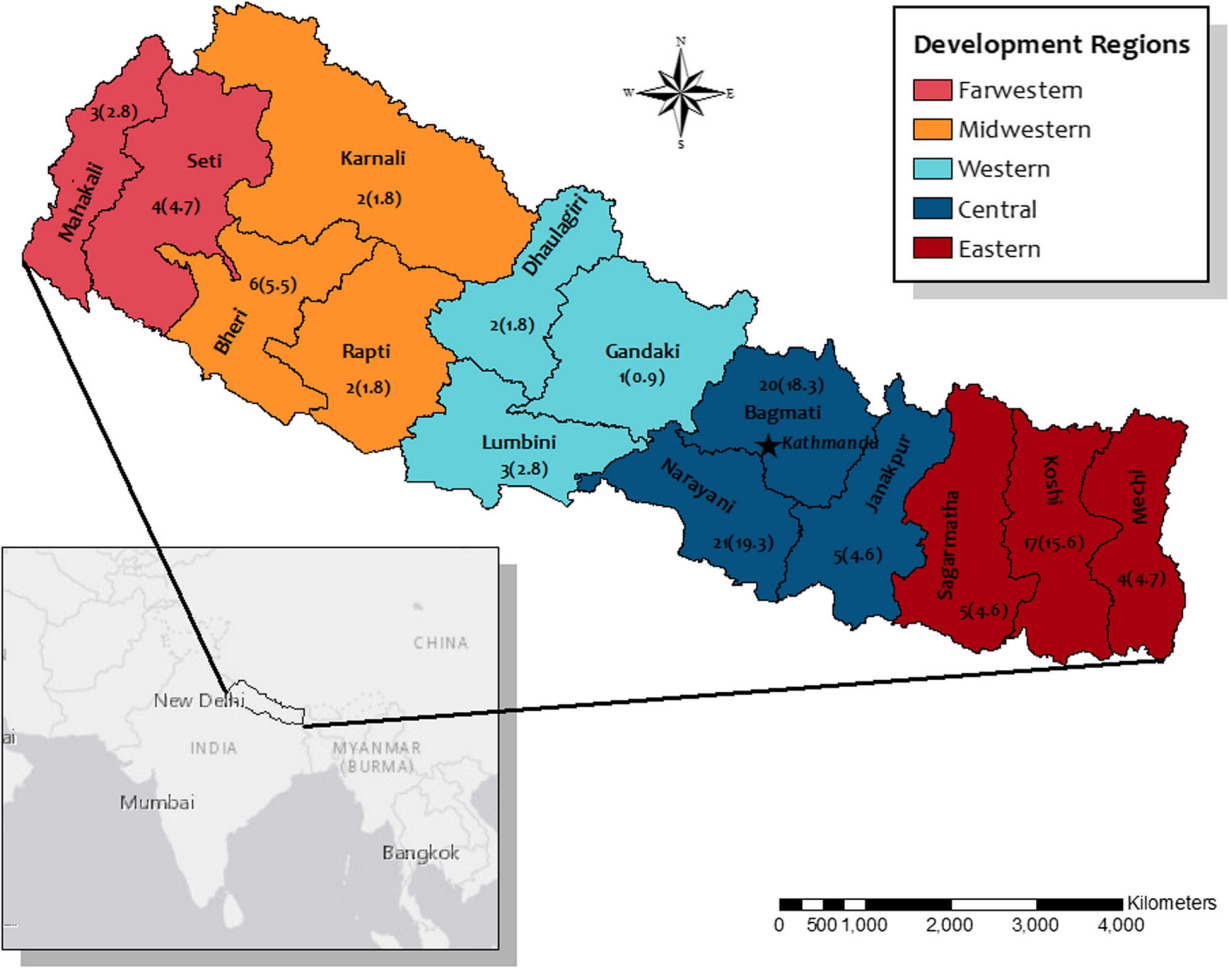
Spatial distribution of health event reports in Nepal by administrative zone*. *Reports do not equal 108 as some reports occurred in multiple zones or the zone was not specified

All covariates differed significantly by source nationality of the health report (Table 1). Compared to health reports from international sources, domestic reports more often reflected active or current health events (*p* < 0.026), were more often reported in newspaper-type media outlets (*p* < 0.0001), and covered events with more focus on human health (p < 0.0001). Regardless of source nationality, health reporting most frequently (*n* = 47, 43.5%) covered events from the Central and Eastern development regions (Fig. 1), which corresponds to Nepali regions with the highest population density and most developed infrastructure. The two main newspaper sources of reports were the *Himalayan Times* (*n* = 25; 23.1%) and *República* (*n* = 32; 29.6%)*,* and both newspapers have a significant online presence in the English language. Primary inter-governmental agency sources (*n* = 12; 11.1%) included the Food and Agricultural Organization, United Nations, and the World Organization for Animal Health (OIE), as well as the Public Health Agency of Canada. A physician posting on the global electronic reporting system for outbreaks, ProMED-mail, submitted the individual contributions (*n* = 4; 3.7%). Poultry trade websites from Asia were the source of reports relating to some of the avian influenza events in-country (*n* = 3; 2.8%). No reports from government agencies nor trade websites were noted from domestic sources during the study period. Domestically, reports most often described events that primarily impacted humans (*n* = 41; 50%), followed by those involving both humans and the environment (*n* = 31; 37%), such as cholera outbreaks and hepatitis E. Internationally, events that primarily affected animals only were most often reported (*n* = 14; 56%), with reporting of human-environment events (*n* = 6; 24%) and human-only (*n* = 5; 20%) events the next most common.

**Table 1:** Event media reporting characteristics of unique reports by source origination, October 2013–December 2014 (*n* = 108)

Media source	Domestic (*n* = 83)	International (*n* = 25)	*P*-value (Fisher’s exact)
Categorical variables; no. (%)			
Nature of event			0.026
Informational	17 (21)	7 (28)	
Warning	1 (1)	3 (11)	
Active	65 (78)	15 (58)	
Development region			0.022
Farwestern	7 (8)	0 (0)	
Midwestern	7 (8)	1 (4)	
Western	7 (8)	0 (0)	
Central	33 (40)	11 (42)	
Eastern	21 (26)	5 (19)	
Multiple regions	3 (4)	0 (0)	
Unspecified regions	5 (6)	8 (32)	
Topic type			<0.0001
Human only	41 (50)	5 (20)	
Animal only	8 (10)	14 (56)	
Environment only	0 (0)	0 (0)	
Human-animal only	2 (2)	0 (0)	
Human-environment only	31 (37)	6 (24)	
Animal-environment only	0 (0)	0 (0)	
Human-animal-environment	1 (1)	0 (0)	
Media type			<0.0001
Newspaper	79 (95)	10 (40)	
Inter-government agency	0 (0)	12 (48)	
Individual (via ProMED)	4 (5)	0 (0)	
Trade website	0 (0)	3 (12)	
Primary group			<0.0001
Humans	74 (89)	11 (44)	
Wildlife	0 (0)	0 (0)	
Livestock and Poultry	9 (11)	14 (56)

When examining characteristics on the event level, domestic sources (*n =* 44, 53%) were more likely to identify a unique event than international sources (*n =* 6, 24%) indicating international sources were more likely to publish multiple reports about the same event (*p* < 0.012). Additionally, more unique events were reported when examining primarily human health events (*n* = 43; 50%), as compared to primarily animal health events (*n* = 7; 32%); though these findings were not statistically significant (*p* < 0.127). However when only focusing on animals, a small number of unique poultry events (*n* = 3) were more likely to be covered in a greater number of reports (*n* = 17) than other animal species. This finding was not surprising given the constant attention provided to avian influenza from international sources.

**Fig. 2:**
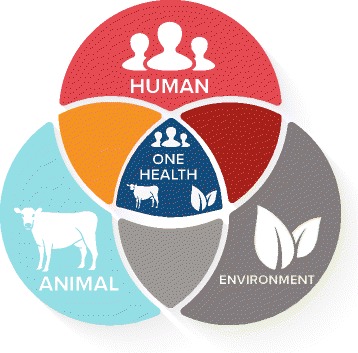
Diagram of health report topics apportioned from a One Health perspective

Collectively, the four most commonly reported health conditions, cholera (*n* = 18; 16.7%), avian influenza (*n* = 17; 15.7%), dengue (*n* = 14; 13.0%), and diarrhea/gastroenteritis not specifically linked to cholera (*n* = 12; 11.1%), accounted for 61% of the reported illnesses (Table 2). Avian influenza, foot and mouth disease, and a suspected case of peste des petits ruminants were conditions only reported in animals, and were also listed as reportable diseases to organizations such as the OIE [28]. Of the conditions specifically linked to humans and the environment only (no animals), cholera, unspecified diarrhea, hepatitis B/E (including jaundice), malaria, and food poisoning were reported. Of note, two of these same conditions, cholera and diarrhea, were likewise reported to involve humans only with no mention in the report of the environment as a factor in disease transmission. No events were reported that involved the environment only or animal-environment only (Fig. 2). Exposure to and overuse of pesticides (type unspecified) was the only health report where the article noted it was an event that could potentially impact humans, animals, and the environment.

**Table 2:** Health conditions reported (confirmed or suspected) in Nepal, October 2013 – December 2014 (*n* = 108)

	Number of unique reports (% of total)
Condition	
Cholera	18 (16.7)
Avian influenza	17 (15.7)
Dengue	14 (13.0)
Diarrhea/gastroenteritis	12 (11.1)
Hepatitis B or E (including jaundice)	10 (9.3)
Leprosy	4 (3.7)
Polio	4 (3.7)
Typhoid (viral fever)	4 (3.7)
Foot and mouth disease	3 (2.8)
Influenza (other than avian)	3 (2.8)
Undiagnosed	3 (2.8)
Fever (unspecified)	2 (1.9)
Food-related toxin	2 (1.9)
Japanese encephalitis	2 (1.9)
Malaria	2 (1.9)
Respiratory illness (including pneumonia)	2 (1.9)
Conjunctivitis	1 (0.9)
Pesticide exposure	1 (0.9)
Mushroom poisoning	1 (0.9)
Peste des petits ruminants	1 (0.9)

## Discussion

Our findings show that few articles from Nepal concerning animal and environmental health were captured by HealthMap’s global digital disease detection platform. This lapse could be due to the online surveillance system’s lack of sensitivity in detecting articles with a focus on these topics or to a severe underreporting of animal health and environmental health events in Nepal’s domestic media. Since the HealthMap data collection process emphasizes both human and veterinary diseases, lack of sensitivity could likely play a role in the limited environmental health reports than animal reports. Additionally, findings showed that international sources were more likely to report events related to animal health and pulled from a greater variety of information sources when reporting animal health events, likely due to requirements for reporting of notifiable diseases set by the OIE [28].

Close examination of multiple articles covering the same outbreak event showed great variation in reporting from a One Health perspective. For example, in the case of Japanese Encephalitis (JE), where two articles reported on the same outbreak, one article made no mention of the important human-animal interface (JE is maintained in an enzootic cycle between mosquito vectors and swine hosts – [29]), while the other article briefly described the of ecological roles of pigs, birds, and mosquitos in the life cycle of the virus causing JE. While this finding may not be surprising based on prior research in Hong Kong and the United States [30], it suggests that there may be opportunities to improve media awareness from a One Health perspective for increased quantity and quality of health event reporting in Nepal, perhaps through formal media training in varying aspects of health event reporting.

In this study, health events that were clearly associated with environmental factors were underrepresented. Moreover, the few reported environmentally-related health events focused on contaminated food or water. Environmental concerns such as land degradation or climate change were not specifically recognized as drivers of health problems in event reports. However, several research studies have shown these to be important environmental drivers directly affecting ecosystem health in Nepal [31–33].

Examining media reports to gain information about in-country health-related conditions has been conducted across several settings [15, 34]. This approach has been shown to complement traditional forms of health surveillance, despite questions concerning reporting characteristics such as specificity and event traceability [35]. The primary strength of this approach and indeed our study was that the data were collected from the HealthMap surveillance system, which reaches a global audience and allows for an integration of information across all sectors. In short, the HealthMap platform is a useful tool for One Health-oriented digital disease detection.

Limitations in this research should be noted. First, this research represents only those events that reached HealthMap’s digital platform [36]. The sensitivity of HealthMap in recognizing health events in Nepal is unknown, but research has shown it to be widely variable based on in-country reporting characteristics [12]. Because only certain languages are supported, there was a likely underreporting of pertinent health articles written specifically in the Nepali language. Findings from this research also indicated there was a high likelihood that health event reports from rural areas in Nepal may not appear on the global disease detection stage as frequently as reports from urban areas, and should be explored further. Additionally, this study did not address the accuracy of the event reports obtained by HealthMap, nor is HealthMap specifically designed to detect reports specifically of environmental events (e.g. excess rainfall, droughts, deforestation). Finally, having a longer catchment period for article inclusion would have certainly added to the number of reports gathered concerning local health events. However, given the length of the catchment period, we do not think this longer time period would have changed the associations observed in reporting characteristics across domestic versus international media sources.

When examining risk communication plans in-country, the relationship between government agencies and news media outlets is clearly critical [37]. One often influences the other, but our data suggest either that Nepali language reporting by the Government of Nepal could not be detected by HealthMap or that the domestic media in Nepal independently reports on health events with far greater frequency. More research is needed to better understand integration and acceptance of health event-based reporting in Nepal, especially by academics, government officials, public health agencies, health journalists, and other stakeholders. As more users interface with HealthMap’s participatory surveillance methods, which involves individuals submitting relevant health condition reports, impact evaluations could be conducted to examine how users not only feed information into HealthMap, but also how that information is then used in the field or in policy decisions. Furthermore, this crowdsourcing approach could also assist in the better integration of environmental-only reports. As additional users interact with the HealthMap surveillance system, more viable information of local relevance on a broad range of health-related topics concerning humans, animals, and the environment could become available for enhanced digital disease detection on a global scale.

## Conclusion

It is important to have a better understanding of the characteristics and factors associated with health event reporting in Nepal in order to highlight potential gaps in media coverage on both the domestic and international stage from a One Health perspective. Additionally, understanding the dynamics in one country may better inform researchers on successful risk communication methods through the media in similar countries, which would indirectly aid in the strengthening of a global early warning system. Tracking health events is important not only because it provides recognition of disease emergence, but also because it could offer insight into the success of interventions, such as examining the impact of response during a natural disaster or a disease outbreak.

## Abbreviation

OIE: World Organization for Animal Health
